# Do the elderly have a voice? Advance care planning discussions with frail and older individuals: a systematic literature review and narrative synthesis

**DOI:** 10.3399/bjgp13X673667

**Published:** 2013-09-30

**Authors:** Tim Sharp, Emily Moran, Isla Kuhn, Stephen Barclay

**Affiliations:** University lecturer, Primary Care Unit; Department of Public Health and Primary Care, University of Cambridge, Cambridge.; CLAHRC End of Life Care Group; University lecturer, Primary Care Unit; Department of Public Health and Primary Care, University of Cambridge, Cambridge.; University of Cambridge Medical School Library, School of Clinical Medicine, Addenbrooke’s Hospital, Cambridge.; University lecturer, Primary Care Unit; Department of Public Health and Primary Care, University of Cambridge, Cambridge.

**Keywords:** advance care planning, conversations, elderly, end of life care, frail, systematic review

## Abstract

**Background:**

Recent years have seen marked improvements in end-of-life care, however concerns have been expressed that services are focused on the needs of patients with cancer. This review focuses on conversations about end-of-life care with frail and older people who have no main overriding diagnosis who are estimated to account for around 40% of deaths.

**Aim:**

To investigate the attitudes of the public and healthcare professionals to advance care planning discussions with frail and older people.

**Design and setting:**

Systematic literature review and narrative synthesis.

**Method:**

Articles that related to frail or older individuals and either advance care plans or discussions on end-of-life care were included. Studies of specific conditions or that focused on prognosis, capacity, or resuscitation decisions were excluded.

**Results:**

While a significant minority of frail older individuals would find them unwelcome, the majority would appreciate the chance to discuss end-of-life care, yet most do not have this opportunity. Attitudes to the timing of these discussions were variable, but most perceived the risk of leaving them too late. Most doctors believed it was their professional responsibility to initiate discussions, but felt limited by time pressures and the absence of a precipitating event. A wide range of barriers were identified including the reluctance of family members to discuss end-of-life care, the passive expectation that someone else would decide on an individual’s behalf, and significant uncertainty concerning future illness and decline.

**Conclusion:**

The marked disparity between the majority of older individuals who would like the opportunity to discuss their end-of-life care and the minority that currently have this opportunity raises important questions if the wishes of this large group in society are to be respected. The challenge is to find effective ways of encouraging dialogue and choice within the constraints of the current healthcare systems and personal circumstances.

## INTRODUCTION

The support people receive towards the end of their lives is being increasingly recognised as an important component of high quality health and social care. In the UK the recent intense pressure to review and the subsequent decision to phase out the Liverpool Care Pathway illustrates the importance the public place on end-of-life care. The well documented phenomenon of people living longer with a greater prevalence of frailty and multiple conditions,^1^ has resulted in a growing population requiring increasingly complex support.

Recent years have seen marked improvements in palliative and end-of-life care. In the UK the Gold Standards Framework (GSF) was developed in 2000 to improve palliative care in primary care. Over 90% of UK GP practices now have a register of patients approaching the end of life. However, these registers are far from comprehensive: only 27% of all patients who died were included in the register before death, of whom 77% had cancer,^2^ despite only 25% of UK deaths being from malignant disease.^3^ As a result concerns continue to be expressed that end-of-life services are focused on the needs of patients with cancer.^4^

In 2008 the UK End of Life Care Strategy^5^ called for open discussions between healthcare professionals and patients approaching the end of their lives as the first step to ensure well-planned care is delivered. It recognised these discussions have many different forms, may be initiated in a broad range of circumstances and should not be the remit of one professional group alone. Patient knowledge that death is approaching and of what can be expected is seen as a prerequisite of a ‘good death’.^6^ In the US the 1990 Patient Self-Determination Act requires health professionals to provide patients with information concerning their decision-making rights and advance healthcare directives on admission to hospital.

This review focuses on conversations about end-of-life care with frail and older people who have no overriding diagnosis. They are estimated to account for around 40% of deaths^7^ and are often associated with multiple comorbidities and a degree of cognitive impairment. Prognostication in this group is very difficult. For those with the frailty of old age, the dying trajectory is more unpredictable than the clearer trajectory of malignancy.^8^

## METHOD

The aim was to undertake a systematic review and narrative synthesis of the literature concerning the attitudes of the public and healthcare professionals to discussions about end-of-life care with frail and older individuals with no overriding diagnosis. The research questions were:

1. Are discussions being held?
2. What are individuals’ attitudes to discussions?
3. What are individuals’ preferences to timing of discussions?
4. What are healthcare professionals’ attitudes to discussions?
5. What are healthcare professionals’ attitudes to timing of discussions?
6. What are the barriers to and facilitators of discussions?

**How this fits in**

This is the first known systematic literature review to look at the attitudes of the public and healthcare professionals to advance care planning discussions with frail and older people towards the end of their life. It found that although a significant minority would find end-of-life care conversations unwelcome, a majority of this growing population would appreciate the chance for such a discussion with healthcare professionals but only a minority have the opportunity. This is despite doctors seeing these conversations as part of their professional responsibilities. The review identified barriers to end-of-life care conversations with frail older patients that were not found in studies of other populations including the reluctance of family members to discuss end-of-life care, the passive expectation that others would decide on their behalf, and the significant uncertainty concerning future illness and decline were particular barriers in frail older individuals. The paper discusses the issues connected with healthcare systems, individual autonomy and personal circumstances that will need to be addressed if the care wishes of this important group in society are to be respected.

An electronic literature search of Medline, CINAHL, PsychINFO, and ASSIA databases from January 1991 to September 2012 was undertaken to cover published research in health and social science. The challenge of developing appropriately sensitive and specific search terms for ‘frail elderly’ with no overriding medical condition was supported by an information officer. The second stage of the search sought articles that either included terms for ‘advance care planning’ or that mentioned words synonymous with both ‘end of life’ and ‘conversations’ or ‘discussions’. Box 1 outlines this search strategy used for the Medline database. Appendix 1 details all search terms used for each database.

**Box 1. Medline search terms**

frail or elderly or ‘frail elderly’ or seniors or ‘senior citizen*’ or elder* or older

**Table:** 

	AND	
‘advance* care plan*’ or ‘advance* directive*’ or exp patient care planning/ or ‘anticipatory care’ or ‘preferred place of care’	OR	end of life’ or ‘end-of-life’ or palliative or terminal
		AND
		discuss or discussions or conversation* or exp decision making/ or exp treatment refusal/

**Figure 1:**
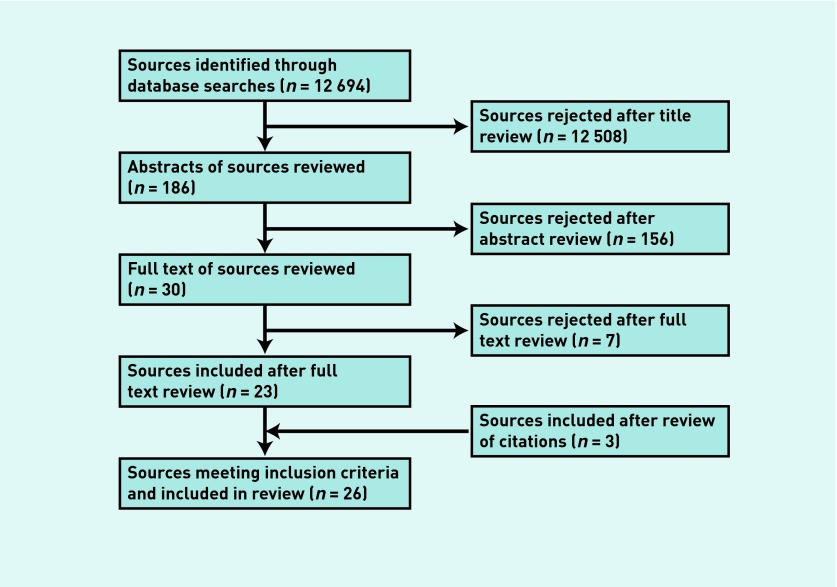
Literature search flow chart.

Exclusion criteria included studies of participants with specific conditions such as cancer, heart failure, chronic obstructive pulmonary disease (COPD), and dementia. Although many of the frail and older individuals will have such conditions, studies of those with a single overriding diagnosis have been excluded. Also excluded were studies that focused on prognosis, capacity or resuscitation decisions and studies not originally published in English. Appendix 2 details the full list of exclusion criteria. The electronic database search generated 12 694 titles which were screened twice to identify potentially relevant papers. One hundred and eighty-six abstracts were reviewed independently. The review protocol was applied and agreement reached on 30 papers to be read in full, seven of which were excluded. A citation search of the 23 included papers identified a further three for inclusion: the final 26 articles were from 20 publications, five from *Archives of Internal Medicine*: no other journal had more than two included papers. The flow chart at Figure 1 shows the distillation to 26 articles. Although there were no geographical criteria, all of the included papers are from US or UK studies.

Data relevant to the review questions was then extracted from these 26 included papers into a study-specific data extraction sheet. Initially double data extraction was undertaken independently on six papers, and standardisation of analysis procedures was ensured. The remaining data extraction was undertaken by the lead author, who led work on the narrative synthesis of the data from each paper in discussion with the review team.

Each included paper was weighted using Gough’s ‘Weight of evidence criteria’.^9^ This includes an attempt to assess the risk of bias within individual studies. The weightings of each paper are shown in the final column of Appendix 3, with the overall weighting given for each study in bold.

## RESULTS

The results of the systematic review are analysed by research question with the number of papers addressing each question given in brackets (*n*). Appendix 3 presents a full list of the papers included in this review, including citation, sample, key findings, and the weighting given to each paper.

### Are end-of-life care discussions being held? (*n* = 16)

Seven papers found between 2% and 29% of frail older people had discussed some form of end-of-life care plans with a healthcare professional.^10^–^16^ The highest proportion was from a study of 600 people admitted to US nursing homes between 1990 and 1994: before the introduction of the Patient Self Determination Act (PSDA) 20% had a discussion of future treatment wishes documented in their notes: after the PSDA this rose to 38%.^10^

The disparity between conversations being held with family members and with healthcare professionals is marked.^11^,^15^ In one study 28% (24/86) of community dwelling senior citizens had discussed the terminal care they wanted with a family member but only 2% (2/86) had discussed it with their physician.^11^ This was the lowest reported rate of discussions with healthcare professionals and in each case was patient initiated.

A further five papers found between 15% and 66%^17^–^21^ of older people had end-of-life care plans documented in their records. Meanwhile between 40% and 79% of doctors report having discussed end-of-life care preferences with older patients.^22^,^23^,^24^,^25^

### What are older individuals’ attitudes to end-of-life care discussions? (*n* = 15)

The majority of papers reported between 61%^19^ and 91%^24^ of older individuals wanted to discuss their end-of-life care.^11^,^12^,^14^,^16^,^19^,^23^,^24^,^26^,^27^–^30^ Some expressed comfort even enthusiasm^14^ for such conversations. In contrast, three studies^18^,^21^,^31^ found a reluctance to have such discussions: including housebound individuals who preferred to live ‘one day at a time’^21^ or people over 50 years who preferred to postpone making plans until they were older or in worse health.^31^

Older people saw the benefits of discussions to include assurance that their wishes would be respected,^30^ the opportunity to address important issues of care and treatment before becoming cognitively impaired^26^ or physically seriously unwell,^24^ and to assist loved ones in making decisions.^30^ They saw the responsibility of initiating discussions to lie with doctors^12^,^28^ who they want to talk in an honest and straightforward manner.^29^

### What are older individual’s preferences for timing of end-of-life care discussions? (*n* = 9)

Most older individuals wanted discussions sooner rather than later:^12^,^14^,^26^,^28^,^29^,^32^ perceiving the risk of ‘leaving it too late’^26^ they thought the benefits of early discussions outweighed any discomfort,^29^ though time and information was needed to make decisions.^32^ Some felt that discussions should happen routinely:

*‘Advance care planning discussions should be routine questions such as screenings like mammograms and colonoscopies. When somebody is X years old, discussions should begin.’*^14^

In contrast, three papers reported older individuals would rather defer discussions, preferring to ‘cross that bridge’ only when they had to,^21^ when the onset of a debilitating or terminal illness precipitated the need to make plans.^31^,^33^

### What are healthcare professionals’ attitudes to end-of-life care discussions? (*n* = 4)

Most doctors felt these discussions to be an important part of their professional responsibility,^23^,^29^ enabling preferences to be well informed and decisions made in the patient’s best interest.^20^,^23^ Many view discussions to be important with patients who have severe chronic illness (91%) or terminal illness (97%), but fewer (64%) felt these conversations were important with older patients regardless of their health status.^23^ While some doctors do not find these conversations stressful,^23^ others comment on the difficulty with frail older people with multiple comorbidities rather than a clear terminal diagnosis, given the uncertainty over future decline and prognosis.^25^

### What are healthcare professionals’ attitudes to the timing of end-of-life care discussions? (*n* = 4)

There was considerable diversity of opinion. Some thought that discussions should start early, before the onset of serious problems.^28^,^20^ Others describe the lack of a clear threshold event, such as a diagnosis, to prompt discussions leaving them to rely on physical or social cues.^25^ While acknowledging their responsibility to initiate discussions, many feared that early discussions may damage the hope that older people bring to the patient–physician relationship.^29^

### What are the barriers to and facilitators of end-of-life care discussions?

A number of themes emerged from the literature:

#### Families (n = 10)

The most frequently identified barrier to discussions are the families of older frail people. It was felt they were sometimes unwilling to have discussions, to accept that their relative is near the end of their life or wish to protect their loved one from upsetting conversations.^14^,^16^,^20^,^26^,^27^,^34^,^35^ Breakdown in family relationships and lack of close family were further obstacles identified.^17^,^31^,^33^

#### Professional and time limitations (n = 9)

Concerns over healthcare professionals’ proficiency and willingness for end-of-life discussions^20^,^27^,^29^,^35^ and perceived lack of continuity of care and support^23^,^31^ are identified as barriers. Some physicians describe being uncomfortable with the ‘paradox of promoting health and discussing its inevitable failure’.^29^ Health professionals also reported the pressure to see a large number of patients and difficulty of scheduling timely follow-up visits conflicts with the time needed for these conversations and so greatly reduced their ability to hold them.^14^,^22^,^23^,^25^,^27^

#### Patient reluctance to discuss (n = 8), feeling ‘others’ would decide (n = 4)

Older frail individuals were found to sometimes be unwilling to discuss their end-of-life care^17^,^20^, ^21^,^24^,^25^,^27^,^31^,^33^ not wanting to talk about such ‘upsetting’^21^ and ‘negative’^17^ issues, not feeling ‘ready to do it’,^21^ or wanting to put off discussions to a time ‘if I ever have a terminal illness’.^33^ They sometimes saw end-of-life care discussions as the responsibility of others, commonly family members.^26^,^33^ Some reported feeling content to leave such matters ‘in God’s hands’,^18^ or that ‘my doctor will decide for me’.^18^

#### Difficulty planning for uncertain future (n = 5). Dementia/lack of capacity (n = 4)

The problems of unforeseen medical scenarios and the difficulty of making well-informed decisions before illness occurs were felt to inhibit end-of-life care planning.^16^,^20^,^21^,^26^,^33^ While cognitive impairment and a lack of decision making capacity were felt to be important barriers to planning.^20^,^27^,^31^,^35^ The onset of dementia was identified as a prompt for early planning.^31^

#### Administrative barriers (n = 4)

A lack of information, inadequate time to consider decisions and the legalistic paperwork involved in completing advance care plans were all felt to be off-putting.^16^,^17^,^29^,^32^

## DISCUSSION

### Summary

Important key themes emerge from this review. A minority of frail and older individuals had end-of-life care conversations with a healthcare professional. Most would welcome the opportunity for such discussions, although a significant minority would find them unwelcome. The preferences for timing are highly variable. The few studies that have investigated healthcare professionals’ attitudes report that doctors see these conversations as part of their professional responsibility, although workload pressures and uncertainty over prognosis inhibited healthcare professionals initiating these discussions.

This review identified important barriers to end-of-life care conversations with the frail and older individuals that were not found in previous reviews. The reluctance of family members to discuss end-of-life care was the most common barrier while the passive expectation that family members, God, or others would decide on their behalf and the significant uncertainly concerning future illness and decline were particular barriers in frail older people.

### Strengths and limitations

Frail and older individuals, unlike groups with a single main diagnosis, are a diffuse group that are challenging to target in a literature search. Low specificity generated a large number of titles to screen: good sensitivity is evidenced by the few additional papers identified by citation searching. The explicit exclusion of older people with a single main diagnosis such as cancer or heart failure, and the focus on those with the multi-comorbidity frailty of old age represents a subset of the older population approaching the end of life: a subset of increasing prevalence as the population ages. Study quality was variable: most were small scale qualitative studies whose primary focus was other than that of this review. The ‘weight of evidence’ scoring 9 of all included papers was thus either low or medium: no paper achieved a high rating. There is a risk of publication and selective reporting bias by parties with a policy agenda, there wasn’t the capacity to undertake a grey literature search to help eliminate this risk.

### Implications for research and practice

The frail and elderly are an important and growing population with challenging care needs. That most would like the opportunity to discuss their end-of-life care but few currently have this opportunity is a marked disparity that raises important questions if the wishes of this large group in society are to be respected.

The issues raised by this review would benefit from further larger-scale studies and in settings other than the US and UK. It would seem reasonable to consider how more can be done to understand the advance care wishes of the frail and older individuals.

The challenge for policy makers and healthcare practitioners is to find effective ways of encouraging dialogue and choice within the constraints of the current healthcare systems and individuals’ circumstances.

#### Healthcare system issues

Healthcare systems across the world have different approaches to how to respond to the needs of the frail and older individuals but the significant pressure on professionals’ time is common. It is therefore not difficult to understand how advance care planning conversations are overlooked, especially when the future is uncertain, there’s no precipitating event and some individuals may find these discussions unwelcome. Healthcare professionals are likely to need support and encouragement to find appropriate opportunities to initiate these discussions. Moreover, increasing expectations of choice among the frail and older individuals, although not necessarily increasing cost, could well have implications for healthcare resource allocations.

- Should professionals have more support and encouragement to initiate these conversations?
- Is there an optimum time to hold these discussions? At a particular age? Or health event (for example, hospital admission or new diagnosis)?
- What are the potential cost and resource implications of more end-of-life discussions?

#### Individual autonomy issues

As well as professionals initiating them, advance care conversations could be promoted as a right, which individuals are encouraged to seek for themselves when they are in good health. Where advanced care planning issues are raised individuals need information and support to help them make informed decisions and these need to be documented and communicated so an individual’s wishes are respected. However, it is important that individuals feel able to refuse such a conversation and don’t feel obliged to make a particular decision or infer that they will receive lower levels of care.

- How can it be ensured that individuals have all the information and time they need to make decisions?
- Should individuals be encouraged to see these discussions as a right?
- How can it be ensured that decisions made by individuals are documented and respected?
- How can it be ensured that the individual’s right to refuse these conversations are respected?

#### Personal circumstance issues

This review highlighted how the reluctance of family members to have end-of-life discussions can act as a significant barrier. Uncertainty over the future, a changing prognosis and cognitive impairment can all make planning difficult. Ways of overcoming barriers from personal circumstances need to be explored if individual autonomy is to be preserved.

- To what extent should an individual’s family be involved in these discussions?
- How can discussions reflect medical uncertainty or a changing prognosis?
- Should a diagnosis of dementia be a prompt for discussions?

Given the findings of this review it would be difficult to argue that more shouldn’t be done to understand and promote the wishes of the frail and older people towards the end of their life. In the context of increased pressure on time and resources, policy makers and healthcare practitioners will need to consider the healthcare and individual circumstance issues raised by this review if the personal autonomy of this important group in society is to be promoted.

## Open access

This is an Open Access article distributed in accordance with the CC BY-NC 3.0 license http://creativecommons.org/licenses/.

## Appendix 1 Search terms

**Four databases; Medline, CINAHL, PsychINFO and ASSIA, were searched between January 1991 to September 2012. Below are the search terms used for each database.**

**Table:**

**Medline search terms**		
frail or elderly or “frail elderly” or seniors or “senior citizen*” or elder* or older		
	AND	
“advance* care plan*” or “advance* directive*” or exp patient care planning/ or“anticipatory care” or “preferred place of care”		“end of life” or “end-of-life” or palliative or terminal
	OR	
		AND
		discuss or discussions or conversation* or exp decision making/ or exp treatment refusal/
**CINAHL search terms**		
frail OR elderly OR “frail elderly” OR seniors OR “senior citizen*” OR elder* or older		
	AND	
“advance* care plan*” or “advance* directive*” or exp patient care plans/ or “anticipatory care” or “preferred place of care”		“end of life” or “end-of-life” or palliative or terminal
	OR	
		AND
**PsychINFO search terms**		discuss or discussions or conversation* or exp decision making/ or exp treatment refusal
frail OR elderly OR “frail elderly” OR seniors OR “senior citizen*” OR elder* or older		
	AND	
“advance* care plan*” or “advance* directive*” or exp patient care plans/ or “anticipatory care” or “preferred place of care”		“end of life” or “end-of-life” or palliative or terminal
	OR	
		AND
		discuss or discussions or conversation* or exp decision making/ or exp treatment refusal
**ASSIA search terms**		
frail OR elderly OR “frail elderly” OR seniors OR “senior citizen*” OR elder* or older		
	AND	
“advance* care plan*” or “advance* directive*” or SU.EXACT.EXPLODE(“Care plans”) or “anticipatory care” or “preferred place of care”		“end of life” or “end-of-life” or death or dying or palliative or terminal
	OR	
		AND
		discuss or discussions or conversation* or SU.EXACT.EXPLODE(“Decision making”) or “treatment refusal”

## Appendix 2 Exclusion criteria

Articles were excluded from the study if they fulfilled one or more of the exclusion criteria.

### Focus

1 Don’t have a focus on the **frail or elderly**.
2. Don’t consider either **discussions** of end of life plans or **advanced care plans**.
3. Focus on **resuscitation** decisions
4. Focus on **prognosis** or **capacity**
5. Focus on **specific conditions**, for example, malignancy, dementia, heart failure, renal failure, psychotic disorders, or COPD.
6. Focus on **differences** between populations based on ethnic minority, sex, or sexual orientation
7. Focus on **assisted suicide** and **euthanasia**
8. Focus on **symptoms** at the end of life
9. Focus on **admission rates** and/or mortality
10. Focus on **congruence** between wishes and execution
11. Focus on socioeconomic factors **predicting** completion of advance directives

### Types of articles

12. **Opinion pieces**, guidelines or individual case reports
13. **Plans for a study** rather than the study results
14. Evaluations of **local programmes, questionnaires or training**
15. **PhD** submissions

### Publication language and timing

16. Not originally published in **English**
17. Published before **January 1991** or after **September 2012**

## Appendix

**Appendix 3:** Articles included in the review

**First author**	**Citation**	**Sample**	**Aim**	**Research methods used**	**Key Findings**	**Weight of Evidence***
Black^22^	J Gerontol Soc Work 2004;43:13 1–46	29 social workers in 6 hospitals in upstate New York, US.	To examine social workers’ advance directive communication with hospitalised elderly patients	Questionnaire	Social workers play an active role in advance directive discussions They reported the time they spent was inadequate	A) Medium B) Low C) Low Overall: **Low**
Black^27^	Home Health Care Serv Q 2007;26:41–58	27 case managers across Florida, US	To explore case managers perceptions of facilitators and barriers to advance care planning practices.	Focus groups	15/27 case managers indicated clients willingly discussed future care plans Time constraints of case management. Lack of available immediate family. Families were sometimes “in denial”, “unrealistic” “dysfunctional” 16/27 said their proficiency in ACP was an issue. Many said dementia limited communication	A) Medium B) Low C) Low Overall: **Low**
Bradley^10^	J Am Geriatr Soc 1998;46:12 35–41.	600 residents admitted between 1990 and 1994 to 6 nursing homes in Connecticut, US	To measure the frequency nursing home residents discuss with clinicians their wishes for future treatment and to assess the influence of the PSDA.	Review of nursing home medical records	28.5% of residents had at least one discussion of future treatment wishes documented This had increased since the implementation of the PSDA from 20.3% in 1990 to 36.7% in 1994).	A) Medium B) Low C) Medium Overall: **Medium**
Carrese^21^	Brit Med J 2002;324:1 25–7	20 chronically ill housebound patients over 75 from Baltimore US	To understand how elderly patients think about and approach future illness and the end of life.	Semi-structured interviews.	16/20 were reluctant to think about or plan for the future. Many preferred to “cross that bridge” of decision making only when had to Felt planning couldn’t be successful because of uncertainty over the future Some felt matters to be in “good hands”	A) Medium B) Medium C) Medium Overall: **Medium**
Clarke^34^	J Pain Symptom Manag 2010;40:85 7–69	74 people older people, informal caregivers and community group representative s from across UK	To explore older people’s concerns about end-of-life issues.	Focus groups	Only three described having made a living will. One said her family were unwilling to talk Some preferred not to talk to their families about death.	A) Low B) Low C) Low Overall **Low**
Damato^11^	N J Med 1993;90:21 5–20.	86 community-dwelling senior citizens from Jersey City, US	To determine knowledge and interest in advance directives and attitudes towards end of life care.	Questionnaire	28% discussed terminal care with family but only 4% put wishes in writing and only 2/86 discussed with their physician 71% indicated desire to prepare instructions in advance	A) Medium B) Low C) Low Overall **Low**
Froggatt^35^	Palliative Med 2009;23:33 2–8	213 managers of care homes in North West and South West England	To describe current Advance Care Planning practices in English care homes for older people.	Questionnaire and telephone interview	89% recommended advanced care planning Managers identified barriers to discussions include physical problems (62%), dementia (81%), families (51%), staff confidence (66%) and communication (40%). Challenges implementing wishes include other health professionals (GPs, district nurses) & other hospitals.	A) Medium B) Low C) Low Overall **Low**
Gamble^12^	Arch Intern Med 1991;151:2 77–80.	75 elderly people in rural eastern North Carolina	To explore the knowledge, attitudes and behaviour of elderly persons regarding living wills.	Questionnaire	81% would like to discuss end of life care with their doctor but only 11% had. 79% thought these issues should be discussed when a person was well. 20% thoughts these discussions should happen when someone was 50 years.	A) Medium B) Low C) Low Overall: **Low**
Golden^17^	Am J Hosp Palliat Care 2009;26:13–7.	1569 home-bound but nursing home eligible older adults in Florida.	To study the prevalence of specific barriers that prevent home-bound older adults from obtaining advance directives.	Interviews	66.2% had an advance directive Of the 530 who didn’t almost 60% of the barriers consisted of the answer “never thought of it”, “chose not to execute” or “do not want to think about a negative topic”.	A) Medium B) Medium C) Low Overall: **Medium**
Gordon^13^	Arch Intern Med 1999;159:7 01–4.	5117 people aged over 65 years.	To find the proportion of seniors who had been asked about their end of life care preferences by a clinician & had completed an advance directive	Questionnaire	One third reported having an advance directive on file but only 15% talked with a clinician about end of life care preferences.	A) Medium B) Medium C) Medium Overall: **Medium**
Johnston^28^	Arch Intern Med 1995;155:1 025–30.	329 adults age 19 to 94 years 282 resident & 272 practicing physicians at 8 primary care clinics across Eastern and Mid-Western US	To assess the opinions of Primary Care Patients and Physicians on discussions on Advance Directives	Questionnaire	The majority of patients & physicians agreed it is the responsibility of the physician to initiate discussions. 91% agreed that AD should be discussed before patients are extremely ill The patients believed the discussions should occur earlier than the physicians	A) Medium B) Low C) Low Overall: **Low**
Malcomson^14^	J Am Acad Nurse Pract 2009;21:18–23.	20 healthy 60–94 year olds Massachusetts US	To explored the perspective of healthy elders to advance care planning.	Focus groups & questionnaire.	Only 20% reported having an advance care planning conversation Participants expressed comfort even enthusiasm for discussing end of life preferences. Healthcare providers should initiate discussions when a patient is relatively well. Barriers identified include time, an emphasis on treatment and cure and a lack of comfort among family, friends & providers.	A) Low B) Low C) Med Overall: **Low**
Markson^23^	J Am Geriatr Soc 1997;45:39 9–406.	653 physicians across US	Investigates how much experience physicians have had discussing and following advance preferences and how physicians perceive their role in the advance decision making process	Questionnaire	79% had discussed advance preferences with at least one patient 82% felt helping patients chose advance preferences was an important part of their responsibilities Most didn’t feel conversations were particularly stressful	A) Medium B) Medium C) Medium Overall: **Medium**
McCarth^15^	J Geront A-Biol 2008;63:95 1–9.	220 community dwelling elders all over 80 years old US	To describe advance care planning, health care preferences and health perceptions in a very elderly sample.	Interview	69% reported discussing their EOL medical care with someone but only 17% discussed their wishes with a physician or health care provider.	A) Medium B) Medium C) Low Overall:**Medium**
Moore^16^	J Gerontol Soc Work 1999;31:21–39.	20 low income community dwelling senior adults in relatively good health age 58–78 years in New York State US	To explore factors that influence an elder’s decision to complete an advance directive	Interview	Only 1/20 had discussed an advance directive with a doctor. Majority would be comfortable if doctor raised an advance directive during a routine visit 8/20 said family reluctant to discuss issues as didn’t want to recognise decline.	A) Low B) Medium C) Low Overall: **Low**
Morrison^24^	Arch Intern Med 1994;154:2 311–8.	277 residents and attending physicians at a large New York Hospital, US	To determine the impact of five proposed barriers to physicians using advance directives.	Questionnaire	40% of physicians hadn’t discussed advance directives with a patient in the last month Barriers included physicians questions about appropriateness, their lack of understanding, lack of comfort & time constraint.	A) Medium B) Medium C) Medium Overall: **Medium**
Palker^18^	Nurse Pract 1995;20:7–8, 13, 17–8, passim.	104 nursing home residents, South Eastern US	To determine the prevalence of advance directives among residents of a Nursing Home, to identify barriers to documentation and to explore death anxiety.	Review of nursing home records plus interview with 17 residents	52% had as least one advance directive 13/17 didn’t know what an advance directive was & didn’t have one. 4/17 had an advance directive &gave largely practical reasons for preparing it	A) Low B) Low C) Low Overall: **Low**
Pfeifer^29^	J Gen Intern Med 1994;9:82–8.	43 primary care physicians and 47 ambulatory patients in 8 cities across US	To identify primary care patients and physicians attitudes to discussions of end of life medical care.	Interviews.	Patient preference for discussions in an honest & straight-forward manner. Holding discussions & making decisions early outweighed any discomfort over content. Physicians felt it was their responsibility to initiate discussions but were uncomfortable with early discussions citing risks to patient’s hope.	A) Medium B) Medium C) Medium Overall: **Medium**
Samsi^31^	Health Soc Care Comm 2011;19:52–9.	37 adults over 50 years in UK	To explore experiences, opinions and attitudes of older adults living in community in the context of the Mental Capacity Act.	Interviews	Although a large proportion recognised the benefits of planning they were keen to postpone it until they were older, in worse health or it was “more appropriate” Some were unsure they could rely on their GP, reporting not always seeing the same GP	A) Medium B) Low C) Low Overall: **Low**
Schonfeld^25^	Am J Hosp Palliat Care 2012;29:26 0–7.	Primary care physicians at University of Nebraska Medical Centre, Canada	To explore differences between end-of-life conversations with patients /families with multiple co-morbidities versus a single, terminal diagnosis.	Focus groups	46.9% had initiated end of life conversations where diagnosis was unspecified Overwhelming felt EOL conversations more difficult with multiple co-morbidities. Rely on physical and social cues to prompt discussions Lack of clear threshold or prompting event a barrier	A) Medium B) Medium C) Medium Overall: **Medium**
Seymour^26^	Soc Sci Med 2004;59:57–68.	32 individuals from older people’s community groups in Sheffield UK	To explore older people’s views on advance statements and the role these might play in end of life care decisions.	Focus groups	Recognition of opportunity advance statements offered to address care & treatment issues before cognitive impairment. Perceived risk of “leaving it too late” Most envisaged problems making decisions for future situations that were difficult to imagine	A) Low B) Low C) Med Overall: **Low**
Stelter^19^	Arch Intern Med 1992;152:9 54–9.	214 people over 65 years attending senior centres in Midwest US.	To learn the reasons why so few people had completed living wills	Questionnaire	15% had completed a living will. 66% without one had planned to complete one. 94% believe planning for the future is important. 61% wanted to discuss living wills with a physician 84% reported comfortable talking about the end of life	A) Medium B) Medium C) Low Overall: **Low**
Stewart^20^	Age Ageing 2011;40:33 0–4.	Staff & families from 34 homes for older people in London, UK.	To explore views on advance care planning in care homes for older people.	Interviews	Staff felt ACP promoted respect for residents’ wishes and aided their treatment decisions. Discussions should start early, in gradual stages before the onset of serious health problems. Barriers include lack of capacity, unforeseen medical scenarios, and the reluctance of some residents and staff to discuss end of life issues	A) Medium B) Medium C) Medium Overall:**Medium**
White^32^	J Am Acad Nurse Pract 2005;17:14–20.	13 new residents at a long-term care facility who had signed an advance directive in Midwest US.	Explore experiences of residents who had signed an advance directive on admission to a long-term care facility and apply author developed model.	Interviews	Sample had signed an advance directive on moving to care facility, 45% had consulted their physician regarding their advance directive. Need time and information to make decision.	A) Low B) Low C) Low Overall: **Low**
Winland-Brown^33^	Adv Pract Nurs Q 1998;4:36–40.	17 people over 65 with no formal advance directive Florida, US	To understand older people’s reasons for not having formalized their end-of-life decisions.	Interview	Living in & accepting the present valued over fear of death “If I ever have a terminal illness, I’ll think about advance directives.” “In God’s hands” Confidence family will fulfil wishes	A) Low B) Low C) Medium Overall: **Low**
Zronek^30^	JONAS Healthc Law Ethics Regul 1999;1:23–8.	51 people over 60 years with an advance directive prior to hospital admission in Mid-West US	To examine patients’ beliefs and level of understanding of the advance directives they had completed.	Survey	Most felt well informed about advance directives Benefits of advance directives include ensuring wishes will be heard & assisting family in making decisions	A) Medium B) Low C) Low Overall: **Low**

* **Gough’s “Weight of evidence criteria”**

Papers are assessed on four criteria:

1. Coherence & integrity of the evidence in its own terms

2. Appropriateness of form of evidence for answering review question

3. Relevance of the evidence for answering review question

4. Overall assessment of study contribution to answering review question (low, medium or high)

Criteria 1 involved an attempt to assess the risk of bias within individual studies. The weightings of each paper are shown in the final column of Table 1 with the weighting given for overall assessment of study contribution (criterion 4) in bold.
